# 5-ASA induced interstitial nephritis in patients with inflammatory bowel disease: a systematic review

**DOI:** 10.1186/s40001-022-00687-y

**Published:** 2022-04-29

**Authors:** James G. Moss, Christopher M. Parry, Richard C. L. Holt, Stephen J. McWilliam

**Affiliations:** 1 Department of Women’s and Children’s Health, Institute of Life Course and Medical Sciences, University of Liverpool, Liverpool, UK; 2 Institute in the Park, National Institute for Health Research Alder Hey Clinical Research Facility, Alder Hey Children’s Hospital, East Prescot Road, Liverpool, UK; 3 Department of Paediatric Nephrology, Alder Hey Children’s Hospital, Liverpool, UK

**Keywords:** Drug induced acute interstitial nephritis, 5-aminosalycilate, Inflammatory bowel disease

## Abstract

**Background:**

Acute interstitial nephritis (AIN) is an important cause of kidney injury accounting for up to 27% of unexplained renal impairment. In up to 70% of cases, drugs, including aminosalicylates, are reported as the underlying cause. Following two recent paediatric cases of suspected mesalazine induced AIN within our own department, we performed a systematic review of the literature to address the following question: In patients with inflammatory bowel disease (IBD), is interstitial nephritis associated with 5-aminosalicylate (5-ASA) treatment? Our primary objective was to identify the number of cases reported in the literature of biopsy-proven 5-ASA induced interstitial nephritis, in children and adults with IBD. We also aimed to identify which variables influence the onset, severity and recovery of 5-ASA interstitial nephritis.

**Methods:**

Embase and PubMed databases were searched from inception to 07/10/20. Search terms had three main themes: “inflammatory bowel disease”, “interstitial nephritis” and “aminosalicylates”. Studies were included if they reported an outcome of AIN, confirmed on biopsy, suspected to be secondary to a 5-ASA drug in those with IBD. A narrative synthesis was performed.

**Results:**

Forty-one case reports were identified. Mesalazine was the most frequently reported aminosalicylate associated with AIN (95%). The median duration of treatment before AIN was diagnosed was 2.3 years (Interquartile Range (IQR) 12–48 months). The median rise in creatinine was 3.3 times the baseline measurement (IQR 2.5–5.5). Aminosalicylate withdrawal and steroids were the most frequently used treatments. Despite treatment, 15% of patients developed end-stage renal failure.

**Conclusions:**

AIN is a serious adverse drug reaction associated with aminosalicylates, with mesalazine accounting for most reports. The current guidance of annual monitoring of renal function may not be sufficient to identify cases early. Given the severity of AIN and reports in the literature that early treatment with steroids may be beneficial, we would recommend at least 6 monthly monitoring of renal function.

*PROSPERO registration number* CRD42020205387.

**Supplementary Information:**

The online version contains supplementary material available at 10.1186/s40001-022-00687-y.

## Background

Acute interstitial nephritis (AIN) is an important cause of kidney injury, causing up to 27% of cases of unexplained renal impairment in adults [1]. In children it is less common accounting for 3–7% of acute kidney injury (AKI) on biopsy [2]. Causes of AIN include: drugs, infection, systemic inflammatory conditions and idiopathic causes [3, 4]. Drugs are by far the commonest cause accounting for up to 70% of cases [4, 5]. Drug-induced AIN (DI-AIN) occurs in an idiosyncratic and non-dose dependant manner and re-occurs on re-exposure [6, 7]. DI-AIN is felt to be secondary to a non-immunoglobin-E-mediated immune reaction marked by cell-mediated immune injury to the renal tubulointerstitium. The onset of DI-AIN typically occurs 7–10 days after drug exposure, however considerable variation is reported and it may occur much later [6, 7]. Cases of AIN can often be missed due to non-specific symptoms. The extra renal features and classical triad of eosinophilia, fever and rash may only be observed in 1–3% of patients [5, 8]. In children these non-specific symptoms can also be different, with anorexia, malaise and vomiting being the most commonly reported symptoms [9]. Therefore, in cases of unexplained AKI, AIN should be considered with renal biopsy being the diagnostic gold standard. The treatment for DI-AIN is discontinuation of the offending drug, and steroids are also frequently used. Despite mixed evidence for corticosteroid use [5, 10–12], some report greater recovery in renal function with early administration [8, 10]. The degree of renal impairment at presentation can be severe and up to 40% of patients with AIN may require treatment with dialysis [3]. Complete recovery of renal function for patients presenting with AIN occurs in approximately 40%, with 45% having partial recovery and 9–13% having no recovery [5, 8].

Mesalazine-induced AIN is widely reported in the literature [13–16]. With up to a third of patients intolerant of sulfasalazine [17], mesalazine is now the preferred aminosalicylate in inflammatory bowel disease (IBD), recommended as standard therapy for treatment of mild to moderately active ulcerative colitis (UC) in both adults and children [18–20]. Its mechanism of action is not fully understood, but some of the ways it is thought to act include: blocking production of pro-inflammatory prostaglandins and leukotrienes, down-regulation of anti-angiogenic factors, blocking production/release of interleukin (IL)-1 and tumour necrosis factor (TNF)-α, inhibitory effect on macrophage and neutrophil function, and promotion of intestinal epithelial wound healing [21].

Both the British Society of Gastroenterology and European Society for Paediatric Gastroenterology, Hepatology and Nutrition recognise that 5-ASA may be associated with renal implications, including the rare idiosyncratic reaction of interstitial nephritis. For patients prescribed regular 5-ASA therapy, it is recommended that renal function, including estimated glomerular filtration rate (eGFR), should be checked before starting treatment, after 2–3 months, and annually long term. Those with impaired renal function should be monitored more closely [19]. This is consistent with the monitoring requirements in the British National Formulary (BNF) and the BNF for children [22, 23] but is less frequent than that recommended by the manufacturers of mesalazine, where the Summary of Product Characteristics (SmPC) for Pentasa® [24], Octasa^®^ [25] and Salofalk^®^ [26] recommend 3 monthly monitoring of renal function/creatinine.

The association of mesalazine induced AIN is not without dispute. A large Cochrane systematic review evaluated the efficacy and safety of 5-ASA preparations in the treatment of mild to moderate UC (7776 patients); although the authors acknowledged case reports of 5-ASA associated AIN, they found no reports of interstitial nephritis in their review [27]. However, Heap et al*.* found 57 patients with histologically confirmed interstitial nephritis in their study of 5-ASA induced nephrotoxicity in IBD. They also suggested a genetic predisposition with an association found in the human leukocyte antigen (HLA) region [13]. Other reviews of the literature by Co et al., Gisbert et al. and Arend et al. also support the association between mesalalzine and AIN [14–16]. There have been 71 suspected cases of mesalazine induced tubulointerstitial nephritis reported to the United Kingdom’s Medicines and Healthcare Regulatory Agency since 1985 [28] and mesalazine has been cited as the fourth most frequently reported drug causing AIN [29].

We recently had 2 cases of AIN in adolescents referred to the paediatric nephrology team by our paediatric gastroenterology colleagues. Both patients were on treatment with mesalazine, one with Crohn’s colitis and another with unclassified IBD. Mesalazine induced interstitial nephritis was suspected in both cases, with AIN proven on biopsy, and both required treatment with steroid therapy. Details for both of these patients are described below in the results section, and in Additional File 1: Table S1,. Following these cases, we performed a systematic review of the literature to address the following question: In patients with inflammatory bowel disease, is interstitial nephritis associated with 5-ASA treatment? Our primary objective was to identify the number of cases reported in the literature of 5-ASA induced interstitial nephritis, proven by biopsy, in children and adults with inflammatory bowel disease. We also aimed to identify which variables influence the onset, severity and recovery of 5-ASA interstitial nephritis.

## Methods

A systematic literature search was conducted using PubMed and EMBASE databases (from inception up to 7th October 2020) for relevant studies using combinations of keywords: ("inflammatory bowel disease" OR IBD OR "Crohn disease" OR "Crohn's disease" OR "ulcerative colitis") AND (Mesalazine OR Olsalazine OR Balsalazide OR Sulfasalazine OR "5 ASA" OR "5 aminosalicylic acid") AND "interstitial nephritis". For EMBASE Emtree subject headings were also used.

Studies were included if they reported an outcome of interstitial nephritis, confirmed on biopsy, which was suspected to be secondary to a 5-ASA drug in patients of all ages (including children) with inflammatory bowel disease. The exclusion criteria were: non-human studies; no diagnosis of inflammatory bowel disease; no evidence of use of a 5-ASA drug; suspected cases of interstitial nephritis NOT confirmed by biopsy; cases of interstitial nephritis felt to be secondary to another cause (disease or drug) as reported by the author; publication type (review articles, conference abstracts, opinion articles, editorials, and those not written in the English language).

A systematic approach was applied for data extraction and synthesis as detailed in the protocol [PROSPERO registration number (CRD42020205387)] [30]. The databases were searched using the above search terms by one reviewer (JM). A list of potential eligible articles was generated independently by two reviewers (JM and CP) based on titles and abstracts screening. Full text review, data extraction and appraisal of articles (Joanna Briggs Institute Critical Appraisal Tools [31]) was also conducted independently by the same two reviewers (JM and CP). Any disagreement was resolved by a third reviewer (SM). For included studies, the following data were extracted: age of the patient, type of IBD being treated, duration of IBD before 5-ASA drug started, type of 5-ASA drug used, daily dose, duration of 5-ASA treatment, concurrent medications, baseline renal function, renal function following diagnosis of interstitial nephritis, treatment of interstitial nephritis and any documented recovery in renal function following treatment. As it was felt likely that the systematic review would mainly identify case reports rather than larger studies, we planned to produce a narrative assessment of the results. Statistcal analysis was performed using Minitab v18. Correlation was assessed using Pearson’s correlation with statistical significance set at *P* ≤ 0.05.

## Results

**Fig. 1:**
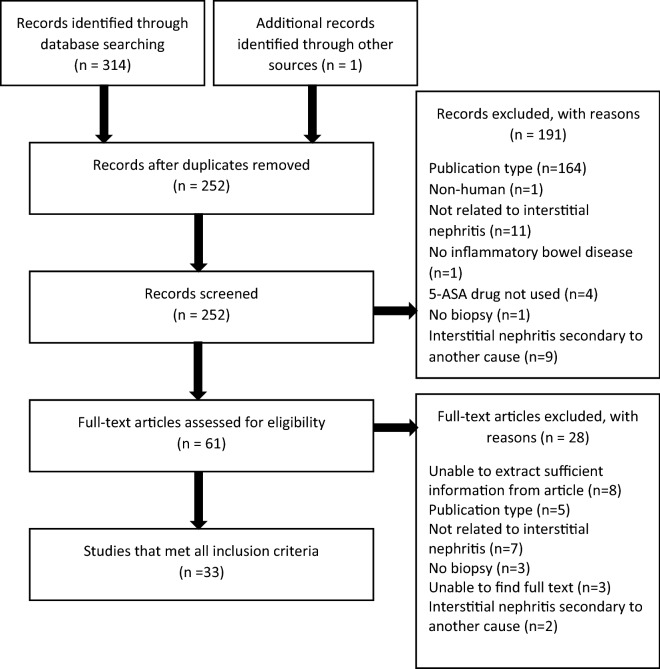
PRISMA flow diagram for literature review

Three hundred and fourteen articles were identified using the search criteria and one additional article was identified through an additional source. Two hundred and fifty-two remained following the removal of duplicates. One hundred and ninety-one were excluded from review of abstracts for not meeting the inclusion/exclusion criteria. Sixty-one articles were reviewed at the full text stage and 33 met our inclusion/exclusion criteria for data extraction. The 28 excluded articles, with reasons, are shown in Fig. 1. The 33 included articles were all case reports, reporting a total of 41 cases.

### Case reports

The most cases reported by a single author was four (Table 1). Cases were published from 1990–2019. Additional File 2: Table S2, shows our data extraction form which includes a full list of the articles identified, data extracted and calculations. Table 2 shows which aminosalicylate was used at the time AIN was reported, the mean daily dose and the median duration of therapy before renal impairment, in months.

**Table 1:** Summary of articles included in systematic review

Authors	Number of cases reported, *n*	JBI checklist score [28]
Clave S., et al., 2019 [32]	1	7/8
Gevorgyan T., et al., 2019 [33]	1	8/8
Lomboy J.R., et al., 2017 [34]	1	8/8
Sato H., et al., 2017 [35]	1	8/8
Vasanth P., et al., 2016 [36]	1	8/8
Magalhaes-Costa P., et al., 2015 [37]	1	8/8
He C., et al., 2013 [38]	1	8/8
Co M.L., et al., 2013 [14]	1	8/8
Gorospe E.C., et al., 2012 [39]	1	7/8
Halbritter J., et al., 2012 [40]	1	8/8
Alivanis P., et al., 2010 [41]	1	8/8
Skalova S., et al., 2009 [42]	1	8/8
Van Biervliet S., et al., 2006 [43]	1	8/8
Tekin F., et al., 2006 [44]	1	8/8
Sari I., et al., 2005 [45]	1	8/8
Tadic M., et al., 2005 [46]	1	8/8
Arend L.J., et al., 2004 [16]	1	8/8
Frandsen N.E., et al., 2002 [47]	1	8/8
Margetts P.J., et al., 2001 [48]	2	8/8
Haas M., et al., 2001 [49]	1	8/8
Benador N., et al., 2000 [50]	2	7/8
Koc M., et al., 2000 [51]	1	8/8
Agharazii M., et al., 1999 [52]	2	8/8
Popoola J., et al., 1998 [53]	2	8/8
Calvino J., et al., 1998[54]	1	8/8
Howard G., et al., 1998 [55]	1	7/8
De Broe M.E., et al., 1997 [56]	1	8/8
Hamling J., et al., 1997 [57]	1	8/8
World M.J., et al., 1996 [58]	4	8/8
Wilcox GM., et al., 1996 [59]	1	8/8
Thuluvath PJ., et al., 1994 [60]	2	8/8
Witte T., et al., 1994 [61]	1	8/8
Mehta R.P., et al., 1990 [62]	1	8/8

**Table 2:** Summary of the aminosalicylate drugs used, including dose and duration of treatment

Aminosalicylate used at time of AIN	Number of cases, *n*	Mean daily dose, g	Median duration of therapy before renal impairment, months
Mesalazine	39	2.2 (*n* = 29)	31.5 (*n* = 34)
Sulfasalazine	1	2 (*n* = 1)	8 (*n* = 1)
Olsalazine	1	0.5 (*n* = 1)	8 (*n* = 1)

*AIN* Acute interstitial nephritis

Twenty-five patients had UC, 12 had Crohn’s disease, 2 were termed as having ‘IBD’, 1 had lymphocytic colitis and 1 had proctocolitis. In most cases, the duration of symptoms before formal IBD diagnosis and commencement on 5-ASA treatment was not known. Of the 6 cases where data was available, the median duration of symptoms was 15 months (IQR 4.5–24 months). The median age of patients at the time of AIN diagnosis was 29.0 years (IQR 19–43 years). There were 8 paediatric cases (< 18 years of age), with a median age at diagnosis of 14.0 years (12.5–15 years). The median duration of treatment with 5-ASA (all forms) before diagnosis of AIN was 27 months (IQR 12–48 months). There was no association between duration of treatment and relative rise in creatinine (Pearson’s correlation 0.055; *p*-value 0.829). There was also no association between the dose and relative rise in creatinine (Pearson’s correlation  − 0.028; *p-*value 0.906). There was no association between the dose and time to develop AIN for the 25/41 patients where necessary data was available (*p*-value 0.371). There was also no association between the age of the patient and the time to develop AIN (Pearson’s correlation  − 0.204; *p*-value 0.505), rise in creatinine (Pearson’s correlation  − 0.204; *p*-value 0.388) and recovery (defined as the overall change in creatinine as a ratio; Pearson’s correlation  − 0.215; *p*-value 0.406).

In all the cases identified, the authors attributed the AIN to the aminosalicylate. In 20 cases, there was documented use of concurrent medications, the most common of which were: prednisolone (*n* = 9), azathioprine or 6-mercaptopurine (*n* = 3), omeprazole (*n* = 2) and amlodipine (*n* = 2).

A baseline creatinine was only recorded in 24 of the 41 cases. The median rise in creatinine was 3.3 times the baseline level (IQR 2.5–5.5).

In the treatment of DI-AIN, 5-ASA withdrawal was documented in 39 cases. In one case [36], mesalazine was not discontinued on the advice of the referring gastroenterologist, due to “financial factors” and the “relative stability of her previously difficult-to-control disease.”

The additional treatments used for the drug induced AIN are shown in Table 3.

**Table 3:** Treatments used in AIN

Treatment	Number of Patients, *n*
Corticosteroids (prednisolone; methylprednisolone)	32
Haemodialysis	4
Azathioprine	4
Mycophenolate mofetil	1
Ganciclovir	1
Amlodipine	1
Labetalol	1
Intravenous hyperalimentation	1
Granulocyte and monocyte adsorption apheresis	1
Withdrawal of drug only	5

Following treatment for AIN, the overall change in creatinine (expressed as a ratio from baseline) was a 1.8 increase (IQR 1.5–2.5). Six patients (14.6%) were left with end-stage renal failure (Chronic Kidney Disease (CKD) stage G5).

### Paediatric studies

Eight of the 41 cases included in this review were children (< 18 years). Three patients had UC, 4 had Crohn’s disease, and 1 had proctocolitis. Six patients were treated exclusively using mesalazine and 2 patients were initially treated with sulfasalazine before switching to mesalazine. The median duration of treatment with 5-ASA was 36.0 months (IQR 14–36 months). The mean daily dose of mesalazine was 2.6 g (Standard Deviation (SD) 1.4 g).

A baseline creatinine was available in 4 of the 8 cases. The median rise in creatinine was 2.7 times the baseline measurement (IQR 1.7–7.0).

Following treatment for AIN, the overall change in creatinine (expressed as a ratio from baseline) was 0.6 (IQR 1.6–2.1); lower than in the adult cases. It is not possible to accurately comment on the degree of renal impairment following treatment for AIN in these paediatric patients as height data were not available to calculate eGFR using the Bedside Schwartz equation, which is currently considered the best method of estimating GFR in children [63]. However, five patients were left with a plasma creatinine value greater than the upper limit of the reference interval for their age group, indicating that these patients developed chronic kidney disease [64].

### Two additional cases from Alder Hey Children’s Hospital

The two cases of mesalazine DI-AIN that prompted this systematic review are detailed in Additional File 1: Table S1, with further clinical and biochemical information. One patient had Crohn’s colitis and the other unclassified IBD. Both patients were prescribed a total daily dose of 3 g of mesalazine and had been taking the drug for at least 3.3 years. Both patients were known to have a baseline creatinine within the normal range for their age group [64] before diagnosis of DI-AIN. The diagnosis of DI-AIN was suspected based on biopsy findings and the use of mesalazine in both cases. One patient had an 1.8 fold increase in their creatinine and the other a 3.3 fold increase. Each patient received prednisolone, and one received mycophenolate mofetil, as treatment for their AIN. Despite this, each patient was left with some degree of chronic renal impairment (≥ CKD G2) following treatment for their AIN.

## Discussion

The last systematic review looking at 5-ASA associated renal injury in IBD patients was published in 2007 [15]. Our review has identified a further 14 case reports since this publication, indicating that aminosalicylate induced AIN continues to be a concern. We have also included details of two further paediatric cases from our centre. Unfortunately, it is not possible to obtain a precise estimate of incidence or prevalence of DI-AIN in this patient group, as we do not have information on the total number of 5-ASA treated IBD patients nationally or worldwide. Our systematic review was triggered by the two cases that we had identified in our hospital among a prevalent cohort of 102 children with IBD receiving 5-ASA treatment.

Our results show that mesalazine was the aminosalicylate drug prescribed in 95% of cases at the time DI-AIN was diagnosed. Of these, 16.6% were also treated with sulfasalazine before treatment was changed to mesalazine. Only 2 case reports identified a different aminosalicylate as the suspected causative agent for DI-AIN diagnosis, and one of these patients had previously been treated with mesalazine. When reviewing the literature, there are reports supporting mesalazine as the most likely aminosalicylate to be associated with AIN [65, 66] and those that dispute this [67]. It is not possible to say from our review if the greater number of reports for mesalazine are due to mesalazine itself being more likely to induce AIN, or if the more numerous reports are due to mesalazine being by far the most frequently prescribed aminosalicylate for IBD.

Clinicians need to be aware of the need for continued vigilance to detect the onset of DI-AIN, as the onset is rarely just after commencement of 5-ASA treatment and more typically months or years later. In our review, the median duration of treatment with aminosalicylates before AIN was diagnosed was 2.3 years, with a minimum time of 6 days and a maximum time of 17 years (12 years of sulfasalazine treatment followed by 5 years of mesalazine). This is consistent with the median duration of 3 years found in the observational study by Heap et al. [13]. Interestingly, the manufacturers of mesalazine [24–26], recommend more frequent monitoring in the first year of treatment and the BNF and the British Society of Gastroenterology and European Society for Paediatric Gastroenterology, Hepatology and Nutrition recommend renal function is checked 2–3 month after starting treatment. This is not consistent with the median duration of 2–3 years of treatment before AIN is diagnosed. However, other blood parameters should be monitored following the initiation of treatment, such as liver function tests and full blood count. Although most patients who develop AIN will present after several years of treatment, the earliest case identified from our review was 6 days, so if bloods are required anyway, then it would be prudent to also include renal function to detect the cases that may present earlier.

We found no association between duration of treatment or dose on the degree of increase in creatinine. We also found no association with the patients age on the time of onset of AIN, its severity, or its recovery. Due to the small numbers in our analysis it is difficult to confidently conclude that no such association exists.

Given the severity of the renal impairment found (median rise in creatinine was 3.3 times the baseline measurement) and the sometimes irreversible damage caused (14.6% of patients developed end-stage renal failure, CKD stage G5), the current recommendation of annual monitoring may not be frequent enough to identify any deterioration in renal function at an early stage, when intervention may be more likely to result in recovery and preservation of renal function. We would suggest at least 6 monthly monitoring of renal function as more appropriate in those with stable renal function and no risk factors for renal impairment. A recent review by Guillo et al. also recommend at least 6 monthly monitoring of renal function after the first year of treatment or every 3 months if there are comorbidities such as hypertension or diabetes, if there is chronic renal disease, use of nephrotoxic drugs or concomitant steroid therapy [68]. This advice was based on a number of experts groups recommendations [68].

We found no association between the dose and degree of rise in creatinine. The mean daily dose in our review was 2.2 g, which was again similar to that found by Heap et al. [13].

Treatment of DI-AIN involves stopping the offending agent. Our review demonstrated that this occurred in 95% of cases. In one case, there was a conscious decision to continue mesalazine despite this being the drug suspected of causing the DI-AIN; this could be considered a high-risk treatment strategy, given the possibility of development of progressive chronic kidney disease.

The evidence behind the use of steroids in DI-AIN is mixed, but there are reports suggesting early treatment with steroids is beneficial [8, 10]. Although we were not able to establish the timing of steroid administration from our review, the majority (78%) of patients received steroids as part of their treatment. Due to the very low quality of the evidence available, i.e. case reports [69], it is not possible to confidently comment on whether those treated with steroids had better outcomes than those who did not receive steroids. Larger cohort studies are required to investigate this further in order to make any firm recommendations.

Being paediatricians, we were particularly interested in any cases affecting children in our review. Paediatric patients represented 19.5% of the cases found in the literature. Unfortunately, due to height data being unavailable, we were unable to calculate paediatric eGFR by the preferred method. However, there was a similar ratio of plasma creatinine increase from baseline among children at presentation compared to adult cases, suggesting a similar degree of severity. There was also a similar duration of treatment before DI-AIN was diagnosed (2.3 years vs 3 years for adults). The treatment for DI-AIN was similar to adult patients with 87% of children receiving steroids. Overall, paediatric cases showed a greater recovery of renal function when compared to the adult cases identified, although 5 of the 8 paediatric cases (62.5%) had a persisting raised creatinine level after treatment of DI-AIN, indicating that these children may be at risk of progressive chronic kidney disease in later life. This unfortunately was the case for the two additional cases reported from our local centre.

The main limitation of this systematic review is the paucity of case reports with complete clinical and biochemical data available. This is particularly relevant to paediatrics, where renal biopsies occur in much smaller numbers [70]. There is a lack of national and international consensus guidelines on when renal biopsies should be performed in this clinical scenario, [71] which could also contribute to the under-reporting of cases.

## Conclusions

In summary, there are ongoing reports of 5-ASA induced AIN, a rare but serious complication, with mesalazine accounting for the majority of cases. Given the non-specific symptoms associated with DI-AIN, recognition often depends on routine measurement of renal function. Given the severity of renal damage secondary to DI-AIN and the idiosyncratic nature in which it develops we would recommend the routine monitoring of renal function at least bi-annually in patients taking 5-ASA medications with stable renal function and no risk factors for renal impairment.

## Supplementary Information

**Additional file 1: Table S1.** Clinical and biochemical information from the 2 cases of suspected mesalazine induced AIN at Alder Hey Children’s Hospital.

**Additional file 2: Table S2.** Data extraction form, including the extracted data from our systematic review.

## Data Availability

Please see PROPSERO for our published protocol CRD42020205387. Data from the two reported cases at our hospital can be found in Additional File 1: Table S1 and the data extraction form from our systematic review and extracted data can be found in Additional File 2: Table S2.

## Abbreviations

5-ASA: 5-Aminosalycilate
AIN: Acute interstitial nephritis
AKI: Acute kidney injury
BNF: British national formulary
CKD: Chronic kidney disease
DI-AIN: Drug-induced acute interstitial nephritis
eGFR: Estimated glomerular filtration rate
HLA: Human leukocyte antigen
IBD: Inflammatory bowel disease
IL-1: Interleukin-1
IQR: Interquartile range
SD: Standard deviation
SmPC: Summary of Product Characteristics
TNF-α: Tumour necrosis factor-α
UC: Ulcerative colitis
