# Supplementary material for: 5-ASA induced interstitial nephritis in patients with inflammatory bowel disease: a systematic review
Source: Eur J Med Res. 2022 Apr 29;27:61. doi: 10.1186/s40001-022-00687-y (PMC9052675; doi:10.1186/s40001-022-00687-y)
Supplement: Supplementary file 1 — Additional file 1: Table S1. Clinical and biochemical information from the 2 cases of suspected mesalazine induced AIN at Alder Hey Children’s Hospital. [file 40001_2022_687_MOESM1_ESM.pdf]

Case Number	Year AIN diagnosed	Patient Age at AIN diagnosis	Gender (M or F)	Type of IBD	Months of disease before starting 5-ASA?	Which 5ASA?	Daily Dose (g)	Duration of 5-ASA treatment (months)?	Concurrent medications?	Which medication(s)?	Withdrawal of 5-ASA?	Other treatment used for the interstitial nephritis
**One**	2019	13	F	CD	6	Mesalazine	3 g	81	Yes	Adalimumab; vedolizumab; methotrexate	Yes	Prednisolone; Mycophenolate mofetil
**Two**	2018	16	M	IBDU	12	Mesalazine	3 g	40	Yes	Azathioprine; adalimumab	Yes	Prednisolone

Case Number	Baseline creatinine (µmol)	Baseline eGFR	GFR stage before AIN	Peak creatinine (µmol)	Worst eGFR	Increase in creatinine (µmol)	Ratio of increase in creatinine	Deterioration in eGFR	Fold decrease in eGFR	Documented creatinine following treatment for AIN (µmol)	Creatinine above upper limit reference interval for age group	Overall change (ratio) in creatinine from baseline following treatment
**One**	61	98	1	111	54	50	1.8	-44	1.8	76	Yes	1.2
**Two**	65	94	1	213	29	148	3.3	-65	3.2	155	Yes	2.4

Case Number	Documented eGFR following treatment for AIN	Overall decrease (ratio) in eGFR, from baseline, following treatment	Change in creatinine from baseline (following AIN treatment)	Change in creatinine from peak (following AIN treatment)	Change in eGFR from baseline (following AIN treatment)	Change in eGFR from peak (following AIN treatment)	GFR stage following treatment for AIN
**One**	79	1.2	15	-35	-19	25	2
**Two**	40	2.4	90	-58	-54	11	3b

Additional file 1: Table S1: Clinical and biochemical information from the 2 cases of suspected mesalazine induced AIN at Alder Hey Children's Hospital

5-ASA: 5-aminosalycilate; AIN: Acute Interstitial Nephritis; CD: Crohn's Disease; eGFR: Estimated Glomerular Filtration Rate; F: Female; GFR: Glomerular Filtration Rate; IBD: Inflammatory Bowel Disease; IBDU: Inflammatory Bowel Disease Unclassified; M: Male

J G Moss, C M Parry, R Holt, S J McWilliam - 5-ASA induced interstitial nephritis in patients with inflammatory bowel disease: a systematic review.
