# Supplementary material for: 5-ASA induced interstitial nephritis in patients with inflammatory bowel disease: a systematic review
Source: Eur J Med Res. 2022 Apr 29;27:61. doi: 10.1186/s40001-022-00687-y (PMC9052675; doi:10.1186/s40001-022-00687-y)
Supplement: Supplementary file 2 — Additional file 2: Table S2. Data extraction form, including the extracted data from our systematic review. [file 40001_2022_687_MOESM2_ESM.pdf]

**Author**	**Year**	**Title**	**Study Type**	**Number of patients**
Clave S. *et al*	2019	Acute tubulointerstitial nephritis in children and chronic kidney disease	Case series	1
Gevorgyan T. *et al*	2019	Mesalamine induced AIB with necrotizing GIN.	Case report	1
Lomboy J.R. *et al*	2017	Allergic Interstitial Nephritis Masquerading as Pyelonephritis in a Pediatric Patient with Crohn Disease	Case report	1
Sato H. *et al*	2017	Interstitial nephritis associated with ulcerative colitis in monozygotic twins	Case report	1
Vasanth P. *et al*	2016	Interstitial Nephritis in a Patient with Inflammatory Bowel Disease	Case report	1
Magalhaes-Costa P. *et al*	2015	Chronic tubulointerstitial nephritis induced by 5-aminosalicylate in an ulcerative colitis patient: A rare but serious adverse event	Case report	1
He C. *et al*	2013	Acute kidney injury in a girl with ulcerative colitis and cytomegalovirus-induced focal segmental glomerular sclerosis	Case report	1
Co M.L. *et al*	2013	Pediatric case of mesalazine-induced interstitial nephritis with literature review	Case report with literature review	1
Gorospe E.C. *et al*	2012	Mesalazine-induced interstitial nephritis in a patient with ulcerative colitis	Case report	1
J. Halbritter. *et al*	2012	MPA: a treatment option for lymphocytic colitis and mesalamine-induced interstitial nephritis.	Case report	1
Alivanis P. *et al*	2010	Reversal of refractory sulfasalazine-related renal failure after treatment with corticosteroids	Case report	1
Skalova S. *et al*	2009	Mesalazine-induced interstitial nephritis	Case report	1
Van Biervliet S. *et al*	2006	Mesalazine interstitial nephritis presenting as colitis ulcerosa exacerbation	Case report	1
Tekin F. *et al*	2006	Acute tubulointerstitial nephritis due to 5-aminosalicylic acid in a patient with ulcerative colitis and chronic renal failure	Case report	1
Sari I. *et al*	2005	Mesalazine-associated acute tubulointerstitial nephritis in a patient with spondylarthropathy	Case report	1
Tadic M. *et al*	2005	Acute interstitial nephritis due to mesalazine	Case report	1
Arend L.J. *et al*	2004	Interstitial nephritis from mesalazine: Case report and literature review	Case report with literature review	1
Frandsen N.E. *et al*	2002	Acute interstitial nephritis associated with the use of mesalazine in inflammatory bowel disease	Case report	1
Margetts P.J. *et al*	2001	Interstitial nephritis in patients with inflammatory bowel disease treated with mesalamine	Case reports	2
Haas M. *et al*	2001	Acute renal failure in a 53-year-old woman with Crohn's disease treated with 5-aminosalicylic acid	Case report	1
Benador N. *et al*	2000	Interstitial nephritis in children with Crohn's disease	Case reports	2

Koc M. *et al*	2000	5-Aminosalicylic acid associated chronic tubulointerstitial nephritis in a patient with Crohn's disease	Case report	1
Agharazii M. *et al*	1999	Chronic interstitial nephritis due to 5-aminosalicylic acid	Case reports	2
Popoola J. *et al*	1998	Lesson of the week: Late onset interstitial nephritis associated with mesalazine treatment	Case reports	2
Calvino J. *et al*	1998	Mesalazine-associated tubulo-interstitial nephritis in inflammatory bowel disease	Case report	1
Howard G. *et al*	1998	Renal dysfunction and the treatment of inflammatory bowel disease (IBD): a case for monitoring	Case report	1
De Broe M.E. *et al*	1997	5-Aminosalicylic acid (5-ASA) and chronic tubulointerstitial nephritis in patients with chronic inflammatory bowel disease: Is there a link?	Case report	1
Hamling J. *et al*	1997	5-Aminosalicylic acid-associated renal tubular acidosis with decreased renal function in Crohn's disease	Case report	1
World M.J. *et al*	1996	Mesalazine-associated interstitial nephritis	Case reports with literature review	4
Wilcox GM. *et al*	1996	Nephrotoxicity associated with olsalazine	Case report	1
Thuluvath PJ. *et al*	1994	Mesalazine induced interstitial nephritis.	Case reports	2
Witte T. *et al*	1994	Interstitial nephritis associated with 5-aminosalicyclic acid	Case report	1
Mehta R.P.	1990	Acute interstitial nephritis due to 5-aminosalicylic acid	Case report	1

**Author**	**Patient Age (Years)**	**Type of IBD**	**How many months of disease before starting 5-ASA?**	**Which 5-ASA?**	**Daily Dose (g)**	**Duration of 5-ASA treatment (months)?**	**Concurrent medications?**	**Which medication(s)?**
Clave S. *et al*	9.5	Crohns Disease	Not Known	Mesalazine	Not Known	Not Known	Not documented	Not applicable

Gevorgyan T. *et al*	51	Ulcerative Colitis	Not Known	Mesalazine	4.8	48	Yes	Omeprazole
Lomboy J.R. *et al*	13	Crohns Disease	Not Known	Mesalazine	2	12	Yes	Infliximab; ciprofloxacin and ceftriaxone
Sato H. *et al*	51	Ulcerative Colitis	6 months	Mesalazine	1.5	3	Not documented	Not applicable
Vasanth P. *et al*	65	Ulcerative Colitis	Not Known	Mesalazine	Not Known	Not Known	Not documented	Not applicable
Magalhaes-Costa P. *et al*	23	Ulcerative Colitis	Not Known	Mesalazine	1.5	17	Not documented	Not applicable
He C. *et al*	15	Ulcerative Colitis	Not Known	Mesalazine	4.8	Not Known	Yes	6-Mercaptopurine; amlodipine and azithromycin
Co M.L. *et al*	14	Ulcerative Colitis	24 months	Mesalazine	1.5	36	Not documented	Not applicable
Gorospe E.C. *et al*	25	Ulcerative Colitis	Not Known	Mesalazine	1.5	120	Not documented	Not applicable
J. Halbritter. *et al*	61	Lymphocytic Colitis	Not Known	Mesalazine	Not Known	6	No	Not applicable
Alivanis P. *et al*	19	Ulcerative Colitis	Not Known	Mesalazine then Sulfasalazine	2	8	No	Not applicable
Skalova S. *et al*	15	Proctocolitis	Not Known	Sulfasalazine then Mesalazine	3	48	Yes	Azathioprine; prednisolone
Van Biervliet S. *et al*	11	Ulcerative Colitis	Not Known	Mesalazine	1.5	36	Not documented	Not applicable
Tekin F. *et al*	56	Ulcerative Colitis	2 weeks	Mesalazine	1.5	0.2	Yes	Amlodipine; prednisolone
Sari I. *et al*	56	Ulcerative Colitis	Not Known	Mesalazine	2	3	Yes	Meloxicam
Tadic M. *et al*	41	Ulcerative Colitis	Not Known	Mesalazine	1.5	48	Not documented	Not applicable
Arend L.J. *et al*	18	Ulcerative Colitis	Not Known	Mesalazine	1.2	18	Yes	Albuterol
Frandsen N.E. *et al*	18	Ulcerative Colitis	Not Known	Mesalazine	2.4	48	No	Not applicable
Margetts P.J. *et al*	29	Ulcerative Colitis	Not Known	Mesalazine	Not Known	13	No	Not applicable
	48	Crohns Disease	Not Known	Mesalazine	0.8	48	Yes	Estrogen
Haas M. *et al*	53	Crohns Disease	Not Known	Mesalazine	2.4	12	Yes	Prednisolone (short course); omeprazole
Benador N. *et al*	16	Crohns Disease	Not Known	Mesalazine	Not Known	Not Known	Yes	Ibuprofen (stopped)
	14	Crohns Disease	Not Known	Sulfasalazine then Mesalazine	Not Known	14	Not documented	Not applicable
Koc M. *et al*	40	Crohns Disease	4 months	Mesalazine	3	72	Yes	Sulfasalazine
Agharazii M. *et al*	33	Ulcerative Colitis	Not Known	Mesalazine	Not Known	60	Yes	Beta blocker

	32	Ulcerative Colitis	Not Known	Mesalazine	Not Known	36	Yes	Ferrous sulfate
Popoola J. *et al*	38	Ulcerative Colitis	Not Known	Mesalazine	2.4	60	Not documented	Not applicable
	25	Ulcerative Colitis	Not Known	Mesalazine	1.6	12	Yes	Prednisolone
Calvino J. *et al*	22	Crohns Disease	Not Known	Mesalazine	1.2	12	Not documented	Not applicable
Howard G. *et al*	69	Inflammatory Bowel Disease	Not Known	Sulfasalazine then Mesalazine	Not Known	36	Not documented	Not applicable
De Broe M.E. *et al*	20	Ulcerative Colitis	Not Known	Mesalazine	1.5	23	Not documented	Not applicable
Hamling J. *et al*	34	Crohns Disease	Not Known	Sulfasalazine then Mesalazine	3	204	Not documented	Not applicable
World M.J. *et al*	31	Crohns Disease	Not Known	Mesalazine	2.4	42	Not documented	Not applicable
	43	Ulcerative Colitis	Not Known	Mesalazine	2.4	27	Yes	Prednisolone; hydrocortisone enemas; azathioprine
	24	Inflammatory Bowel Disease	Not Known	Mesalazine	2.4	22	Not documented	Not applicable
	30	Ulcerative Colitis	Not Known	Sulfasalazine then Mesalazine	1.6	27	Yes	Prednisolone
Wilcox GM. *et al*	71	Crohns Disease	228 months	Olsalazine	0.5	8	Not documented	Not applicable
Thuluvath PJ. *et al*	28	Ulcerative Colitis	Not Known	Mesalazine	2.4	26	Yes	Prednisolone, hydrocortisone
	24	Ulcerative Colitis	Not Known	Mesalazine	2.4	36	Yes	Prednisolone
Witte T. *et al*	22	Crohns Disease	24 months	Mesalazine	Not Known	Not Known	Yes	Prednisolone
Mehta R.P.	29	Ulcerative Colitis	Not Known	Sulfasalazine then Mesalazine	4 g / 2.4 g	79	Yes	Betamethasone enemas

**Author**	**Withdrawal of 5-ASA?**	**Other treatment used for the AIN**	**Baseline creatinine (µmol)**	**Baseline eGFR (ml/min/1.73m^2)**	**GFR stage before developing AIN**	**Peak creatinine (µmol)**	**Worst eGFR (ml/min/1.73m^2)**	**Increase in creatinine (µmol)**	**Ratio of increase in creatinine**	**Deterioration in eGFR (ml/min/1.73m^2)**	**Fold decrease in eGFR**
Clave S. *et al*	Yes	Prednisolone	Not Known	Not Known	Not Known	112	41	Not Known	Not Known	Not Known	Not Known
Gevorgyan T. *et al*	Yes	Prednisolone	62	94	1	274	17	212	4.4	-77	5.5
Lomboy J.R. *et al*	Yes	None	50	Not Known	1	65	Not Known	15	1.3	Not Known	Not Known

Sato H. *et al*	Yes	Prednisolone; granulocyte/monocyte adsorption	78	72	2	162	31	84	2.1	-41	2.3
Vasanth P. *et al*	No	Prednisolone, Mycophenolate mofetil	79.6	67	2	204.3	23	124.7	2.6	-44	2.9
Magalhaes-Costa P. *et al*	Yes	Steroids; azathioprine	88	98	1	354	20	265	4.0	-78	4.9
He C. *et al*	Yes	Haemodialysis, prednisolone, ganciclovir, amlodipine, labetalol	Not Known	Not Known	Not Known	1618	3	Not Known	Not Known	Not Known	Not Known
Co M.L. *et al*	Yes	Methylprednisolone; prednisolone	71	Not Known	Not Known	1229	Not Known	1158	17.4	Not Known	Not Known
Gorospe E.C. *et al*	Yes	Methylprednisolone; prednisolone	Not Known	Not Known	Not Known	1229	5	Not Known	Not Known	Not Known	Not Known
J. Halbritter. *et al*	Yes	Prednisolone	80	91	1	364	16	284	4.6	-75	5.7
Alivanis P. *et al*	Yes	Methylprednisolone	106	83	2	734	9	628	6.9	-74	9.2
Skalova S. *et al*	Yes	Methylprednisolone; prednisolone	Not Known	Not Known	Not Known	130	69	Not Known	Not Known	Not Known	Not Known
Van Biervliet S. *et al*	Yes	Prednisolone	Not Known	Not Known	Not Known	78	Not Known	Not Known	Not Known	Not Known	Not Known
Tekin F. *et al*	Yes	Prednisolone	195	33	3	557	10	363	2.9	-23	3.3
Sari I. *et al*	Yes	None	Not Known	Not Known	Not Known	557	10	Not Known	Not Known	Not Known	Not Known
Tadic M. *et al*	Yes	Methylprednisolone	92	84	2	549	11	457	6.0	-73	7.6
Arend L.J. *et al*	Yes	Prednisolone	88	103	1	221	36	133	2.5	-67	2.9
Frandsen N.E. *et al*	Yes	Haemodialysis	65	Not Known	Not Known	2177	3	2112	33.5	Not Known	Not Known
Margetts P.J. *et al*	Yes	Prednisolone	Not Known	Not Known	Not Known	247	21	Not Known	Not Known	Not Known	Not Known
	Not documented	Prednisolone	Not Known	Not Known	Not Known	615	7	Not Known	Not Known	Not Known	Not Known
Haas M. *et al*	Yes	Haemodialysis	71	96	1	1353	3	1282	19.1	-93	32.0
Benador N. *et al*	Yes	Prednisolone	106	Not Known	2	371	Not Known	265	3.5	Not Known	Not Known
	Yes	Prednisolone; azathioprine	71	Not Known	1	133	Not Known	62	1.9	Not Known	Not Known
Koc M. *et al*	Yes	Prednisolone	71	84	2	124	44	53	1.8	-40	1.9

Agharazii M. *et al*	Yes	Corticosteroids	Not Known	Not Known	Not Known	451	14	Not Known	Not Known	Not Known	Not Known
	Yes	None	Not Known	Not Known	1	495	13	Not Known	Not Known	Not Known	Not Known
Popoola J. *et al*	Yes	Prednisolone; azathioprine	76	106	1	246	27	170	3.2	-79	3.9
	Yes	Prednisolone	94	90	1	302	23	208	3.2	-67	3.9
Calvino J. *et al*	Yes	Prednisolone	Not Known	Not Known	1	221	26	Not Known	Not Known	Not Known	Not Known
Howard G. *et al*	Yes	None	90	78	2	230	26	140	2.6	-52	3.0
De Broe M.E. *et al*	Yes	Haemodialysis; methylprednisolone	97	91	1	937	7	840	9.6	-84	13.0
Hamling J. *et al*	Yes	Intravenous hyperalimentation	79.6	76	2	159	34	79.6	2	-42	2.2
World M.J. *et al*	Yes	Prednisolone	Not Known	Not Known	Not Known	330	20	Not Known	Not Known	Not Known	Not Known
	Yes	Methylprednisolone, prednisolone	Not Known	Not Known	Not Known	450	13	Not Known	Not Known	Not Known	Not Known
	Yes	Methylprednisolone; azathioprine; prednisolone	Not Known	Not Known	Not Known	610	11	Not Known	Not Known	Not Known	Not Known
	Yes	Prednisolone	Not Known	Not Known	Not Known	500	9	Not Known	Not Known	Not Known	Not Known
Wilcox GM. *et al*	Yes	None	106	63	2	239	25	133	2.2	-38	2.5
Thuluvath PJ. *et al*	Yes	Prednisolone	Not Known	Not Known	Not Known	394	17	Not Known	Not Known	Not Known	Not Known
	Yes	Prednisolone	88	98	1	300	24	212	3.4	-74	4.1
Witte T. *et al*	Yes	Prednisolone	105	81	2	530	13	425	5.0	-68	6.2
Mehta R.P.	Yes	None	Not Known	Not Known	Not Known	210	35	Not Known	Not Known	Not Known	Not Known

**Author**	**Documented creatinine following treatment for AIN (µmol)**	**Overall change (ratio) in creatinine from baseline following treatment**	**Documented eGFR following treatment for AIN (ml/min/1.73m^2)**	**Overall decrease (ratio) in eGFR, from baseline, following treatment**	**Change in creatinine from baseline (µmol) (following treatment)**	**Change in creatinine from peak (µmol) (following treatment)**	**Change in eGFR from baseline (ml/min/1.73m^2) (following treatment)**	**Change in eGFR from peak (ml/min/1.73m^2) (following treatment)**	**GFR stage following treatment for AIN**	**Quality assessment score (Joanna Briggs Institute)**

Clave S. *et al*	Not Known	Not Known	94	Not Known	Not Known	Not Known	Not Known	53	1	Include
Gevorgyan T. *et al*	177	2.9	28	3.4	115	-97	-66	11	4	Include
Lomboy J.R. *et al*	Not Known	Not Known	88	Not Known	Not Known	Not Known	Not Known	Not Known	Not Known	Include
Sato H. *et al*	169	2.2	29	2.5	91	7	-43	-2	4	Include
Vasanth P. *et al*	136.2	1.7	36	1.9	56.6	-68.1	-31	13	3	Include
Magalhaes-Costa P. *et al*	Not Known	Not Known	52	1.9	Not Known	Not Known	-46	32	3	Include
He C. *et al*	88	Not Known	96	Not Known	Not Known	-1530	Not Known	93	1	Include
Co M.L. *et al*	124	1.8	74	Not Known	53	-1105	Not Known	Not Known	Not Known	Include
Gorospe E.C. *et al*	796	Not Known	8	Not Known	Not Known	-433	Not Known	3	5	Include
J. Halbritter. *et al*	125	1.6	54	1.7	45	-239	-37	38	3	Include
Alivanis P. *et al*	80	0.8	116	0.7	-27	-654	33	107	1	Include
Skalova S. *et al*	97	Not Known	96	Not Known	Not Known	-33	Not Known	27	1	Include
Van Biervliet S. *et al*	71	Not Known	Not Known	Not Known	Not Known	-7	Not Known	Not Known	Not Known	Include
Tekin F. *et al*	195	1.0	33	1.0	0	-363	0	23	3	Include
Sari I. *et al*	159	Not Known	42	Not Known	Not Known	-398	Not Known	32	3	Include
Tadic M. *et al*	273	3.0	24	3.5	181	-276	-60	13	4	Include
Arend L.J. *et al*	133	1.5	65	1.6	44	-89	-38	29	2	Include
Frandsen N.E. *et al*	Not Known	Not Known	Not Known	Not Known	Not Known	Not Known	Not Known	Not Known	5	Include
Margetts P.J. *et al*	138	Not Known	42	Not Known	Not Known	-109	Not Known	21	3	Include
	380	Not Known	12	Not Known	Not Known	-235	Not Known	5	5	Include
Haas M. *et al*	106	1.5	60	1.6	35	-1247	-36	57	2	Include
Benador N. *et al*	265	2.5	22	Not Known	159	-106	Not Known	Not Known	Not Known	Include
	97	1.4	98	Not Known	26	-36	Not Known	Not Known	Not Known	Include
Koc M. *et al*	115	1.6	48	1.8	44	-9	-36	4	3	Include
Agharazii M. *et al*	260	Not Known	26	Not Known	Not Known	-191	Not Known	12	4	Include

	320	Not Known	21	Not Known	Not Known	-175	Not Known	8	4	Include
Popoola J. *et al*	170	2.2	42	2.5	94	-76	-64	15	3	Include
	183	1.9	42	2.1	89	-119	-48	19	3	Include
Calvino J. *et al*	142	Not Known	43	Not Known	Not Known	-80	Not Known	17	3	Include
Howard G. *et al*	230	2.6	26	3.0	140	0	-52	0	4	Include
De Broe M.E. *et al*	345	3.5	21	4.3	248	-592	-70	14	5	Include
Hamling J. *et al*	71	0.9	87	0.9	-8.9	-89	11	53	2	Include
World M.J. *et al*	248	Not Known	28	Not Known	Not Known	-82	Not Known	8	4	Include
	308	Not Known	21	Not Known	Not Known	-142	Not Known	8	4	Include
	354	Not Known	20	Not Known	Not Known	-256	Not Known	9	5	Include
	470	Not Known	10	Not Known	Not Known	-30	Not Known	1	5	Include
Wilcox GM. *et al*	168	1.6	37	1.7	62	-71	-26	12	3	Include
Thuluvath PJ. *et al*	Not Known	Not Known	Not Known	Not Known	Not Known	Not Known	Not Known	Not Known	3	Include
	280	3.2	26	3.8	192	-20	-72	2	4	Include
Witte T. *et al*	245	2.3	31	2.6	140	-285	-50	18	3	Include
Mehta R.P.	140	Not Known	55	Not Known	Not Known	-70	Not Known	20	3	Include

Additional file : Table S2: Data extraction form, including the extracted data from our systematic review

5-ASA: 5-aminosalycilate; AIN: Acute Interstitial Nephritis; eGFR: Estimated Glomerular Filtration Rate; GFR: Glomerular Filtration Rate; IBD: Inflammatory Bowel Disease
